# Successful treatment of thoracic myelopathy caused by spontaneous spinal epidural hematoma (SSEH) combined with calcification of the ligamentum flavum (CLF) by posterior percutaneous endoscopic surgery (PPES): A case report

**DOI:** 10.3389/fsurg.2022.1077343

**Published:** 2023-01-11

**Authors:** Hou Lisheng, Zhang Dong, Bai Xuedong, Shi Jinglei, Nan Shaokui, Gao Tianjun, Ge Feng, He Qing

**Affiliations:** ^1^ Senior Department of Orthopedics, The Fourth Medical Center of PLA General Hospital, Beijing, China; ^2^ Senior Department of Traditional Chinese Medicine, The Sixth Medical Center of PLA General Hospital, Beijing, China

**Keywords:** spontaneous spinal epidural hematoma (SSEH), calcification of the ligamentum flavum (CLF), thoracic myelopathy, posterior percutaneous endoscopic surgery, recovery

## Abstract

**Study Design:**

A retrospective case report.

**Objective:**

To report a case who developed deteriorated paraplegia by spontaneous spinal epidural hematoma (SSEH) based on calcification of the ligamentum flavum (CLF) at the T10–11 level, achieved full neurological recovery following posterior percutaneous endoscopic surgery (PPES).

**Summary of Background Data:**

CLF rarely occurs at the thoracic spine, and the symptom usually progress slowly. SSEH is another rare spinal lesion that might progress rapidly and cause emergent severe spinal cord compression syndrome. Coexistence of SSEH and CLF at the same thoracic level was rarely reported in English literature.

**Methods:**

A 65-year-old man presented to our hospital with the complaint of sensorimotor loss on the lower limbs and dysfunction of bladder for 1 day after a progressive weakness and numbness of the lower limbs for 3 months. MR examination found a dorsal protruding mass at the T10–11 level, while computed tomography (CT) found the protruding mass contained scattered calcified deposits. The patient was diagnosed with thoracic CLF. Decompression *via* PPES was carried out to realize bilateral decompression through a unilateral approach.

**Results:**

During the operation, the protruding mass was found to be composed of SSEH and CLF together. After the operation, the patient's neurological function recovered quickly. One week later, the patient could walk by himself. After 3 months, complete neurological function had recovered.

**Conclusion:**

SSEH could develop based on CLF at thoracic level and cause serious neurological dysfunction. PPES might be an advisable method to remove CLF and evacuate SSEH with good clinical results.

## Introduction

Calcification of the ligamentum flavum (CLF) is a rare crystal deposition disease that mainly affects the central portion of the ligamentum flavum (LF) (1, 2), and it rarely occurs in the thoracic spine (3). Spontaneous spinal epidural hematoma (SSEH) is another rare spinal lesion, whose initial presentations are often miscellaneous and atypical and might cause delayed diagnosis and improper treatment (4).

The fact that SSEH and CLF coexisted at the same thoracic level was extremely rare. We managed one such case, who developed subacute SSEH with pre-existing CLF at the T10–11 level. CLF removal and hematoma evacuation *via* percutaneous posterior endoscopic surgery (PPES) achieved satisfactory recovery.

## Case report

A 65-year-old man visited our hospital on 6 May 2019 complaining of sensorimotor loss in two legs and bladder dysfunction for 1 day after experiencing progressive weakness and numbness in two legs for 3 months. About 4 years ago, he was diagnosed with coronary atherosclerosis and unstable angina and has taken aspirin, atorvastatin, and isosorbide mononitrate daily since to reduce thrombotic cardiac risk prophylactically.

A neurological examination found hypoesthesia below the right L1 dermatome and sensation loss below left L1. Muscle strength of left/right was grade 0/0 between L1 and L4 myotomes, 1–2/0–1 between L5 and S1. The laboratory tests were normal.

**Figure 1:**
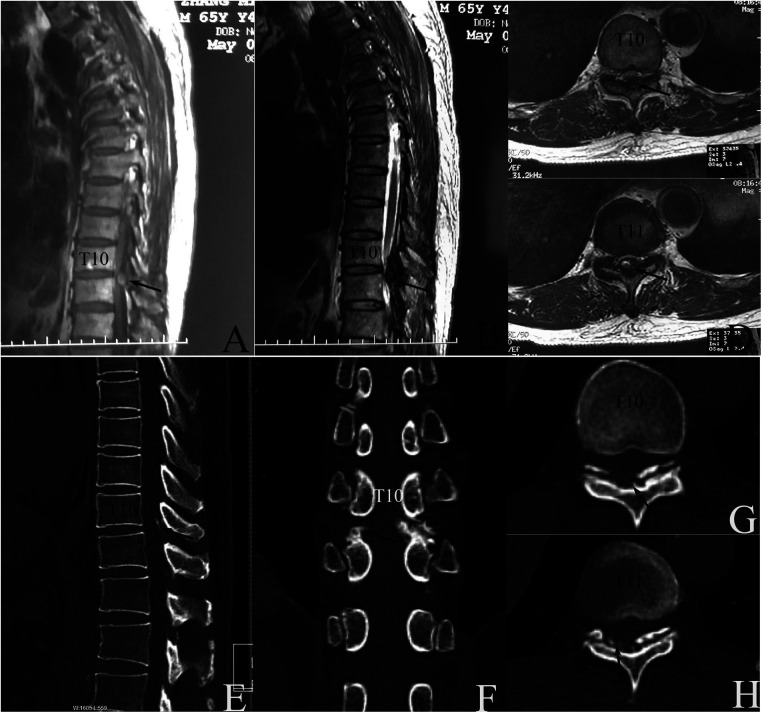
Preoperative image examinations. (**A–D)** Magnetic resonance (MR) taken on 2 May 2019 detected a dorsal epidural compression at T10–11 interlaminar level. (**A**) midsagittal T1WI image; (**B**) midsagittal T2WI image, and transverse T2WI image at T10 (**C**) and T11 (**D**) levels. (**E–G**) Computed tomography (CT) taken on May 6 revealed intraspinal canal calcification deposits posterior to the spinal cord at the T10–11 level which had no continuity with the lamina. Midsagittal (**E**), coronal plane (**F**), and transverse CT image at T10 (**F**) and T11 levels (**G**) on bone window, respectively.

MR examination on 2 May 2019 detected extradural protruding mass compressing the spinal cord dorsally at the T10–11 level with intramedullary hypo-intense signals on T1-weighted images (T1WI) and hyperintense signals on T2-weighted images (T2WI) (Figure 1). The suspected diagnosis included ossification of LF (OLF), CLF, or subacute SSEH with incomplete paraplegia (5).

Subsequent computed tomography (CT) taken on 6 May 2019 found the calcified protruding mass did not attach to the lamina (Figure 1). CLF was diagnosed with the exclusion of SSEH or OLF.

Administration of aspirin was replaced by low molecular weight heparin 3,000 iu injected subcutaneously per day. An intravenous drip of 0.5 g of methylprednisolone succinic acid (MP) for a single time produced temporary neurological improvement.

**Figure 2:**
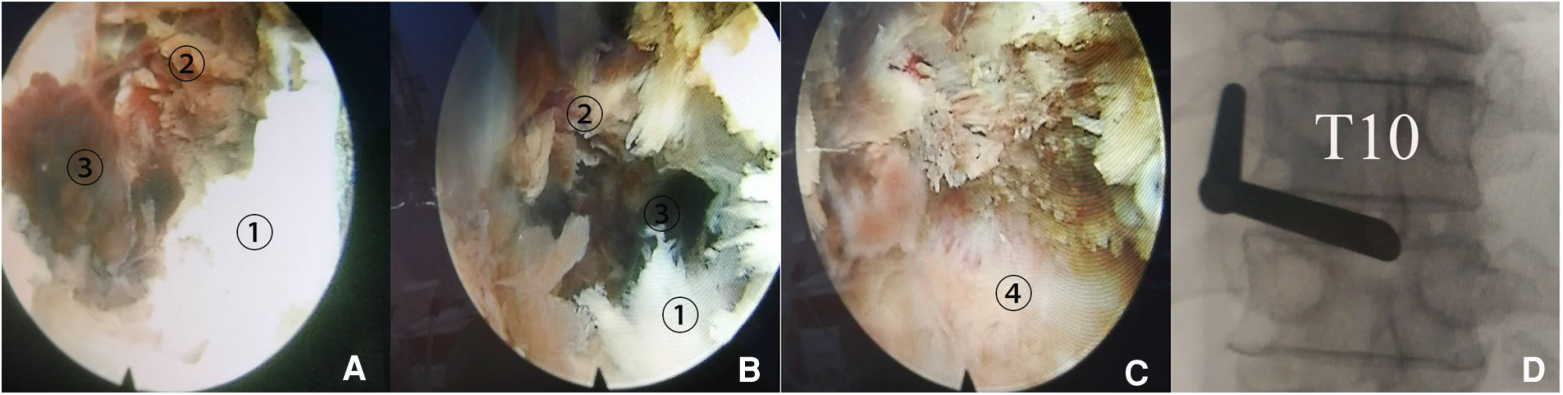
Spontaneous spinal epidural hematoma (SSEH) in addition to calcification of the ligamentum flavum (CLF) compressing spinal cord at the T10–11 level was confirmed intraoperatively. (**A**) Following removal of CLF ①, necrotic deeper layer of ligamentum flavum (NLF) exhibiting purple-red color appeared ②, with stale blood clot in deep-red color squeezed out from opened small mouth of LF 2462. (**B**) SSEH was found to exist between NLF and dural sac. (**C**) Dural sac exposed after evacuation of SSEH. (**D**) Fluoroscopic image confirmed the location of anterior end of working canal.

The decompression operation was postponed and performed 7 days later, on May 13, fearing intraoperative bleeding from aspirin administration (3) above. Bilateral decompression through a unilateral approach was performed using the PPES technique under local lidocaine anesthesia with conscious sedation (6, 7). A high-speed flexible diamond burr (Chongqing Xishan Technology Co., Ltd., Chongqing, China) was used to remove the inferior portion of the left lamina, the base of the spinous process, and the medial aspect of the ipsilateral inferior articular process (IAP) from T10. Then, the posterior layer of LF with its normal white-yellow color was dissected. The deeper LF layer exhibited purple-red color indicating necrotic tissue appeared, meanwhile some dark-red blood clots squeezed out through the opened window of the deeper LF layer with intermittent stale blood fluid draining out. It confirmed intraoperatively that the spinal cord was compressed by SSEH deeply and CLF superficially together. Following removal of bilateral CLF and evacuation of SSEH, the dural sac expanded to its normal elliptical contour and free mobility of the dural sac was visualized endoscopically (Figure 2). No drainage tube was left as there was no obvious bleeding at the end of decompression.

**Figure 3:**
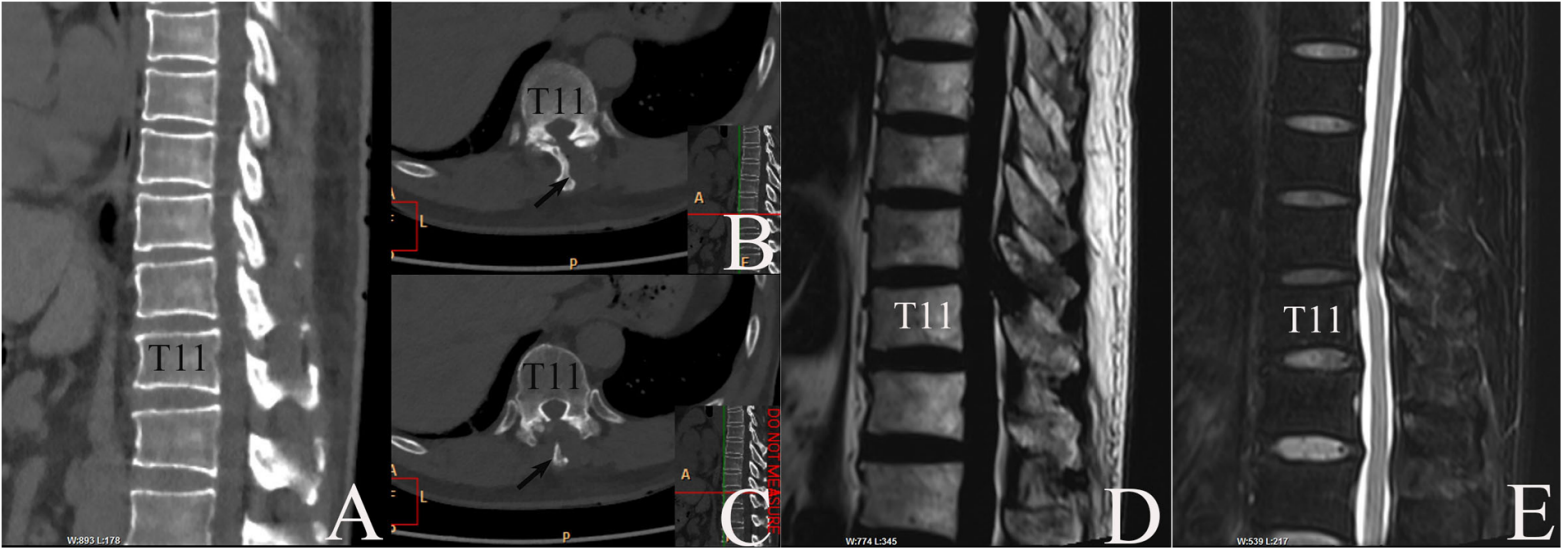
MR and CT re-examinations postoperatively. (**A–C**) CT taken on 15 May 2019 confirmed disappearance of CLF and fenestration at left T10–11 interlaminar space. (**A**) Midsagittal plane, bone window. (**B, C**) Axial plane, bone window. Arrow: T10 spinal process. (**D, E**) MRI taken on 26 December 2019 revealed complete recovery of dura sac. (**D**) Midsagittal T1WI image. (**E**) Midsagittal T2WI image.

**Figure 4:**
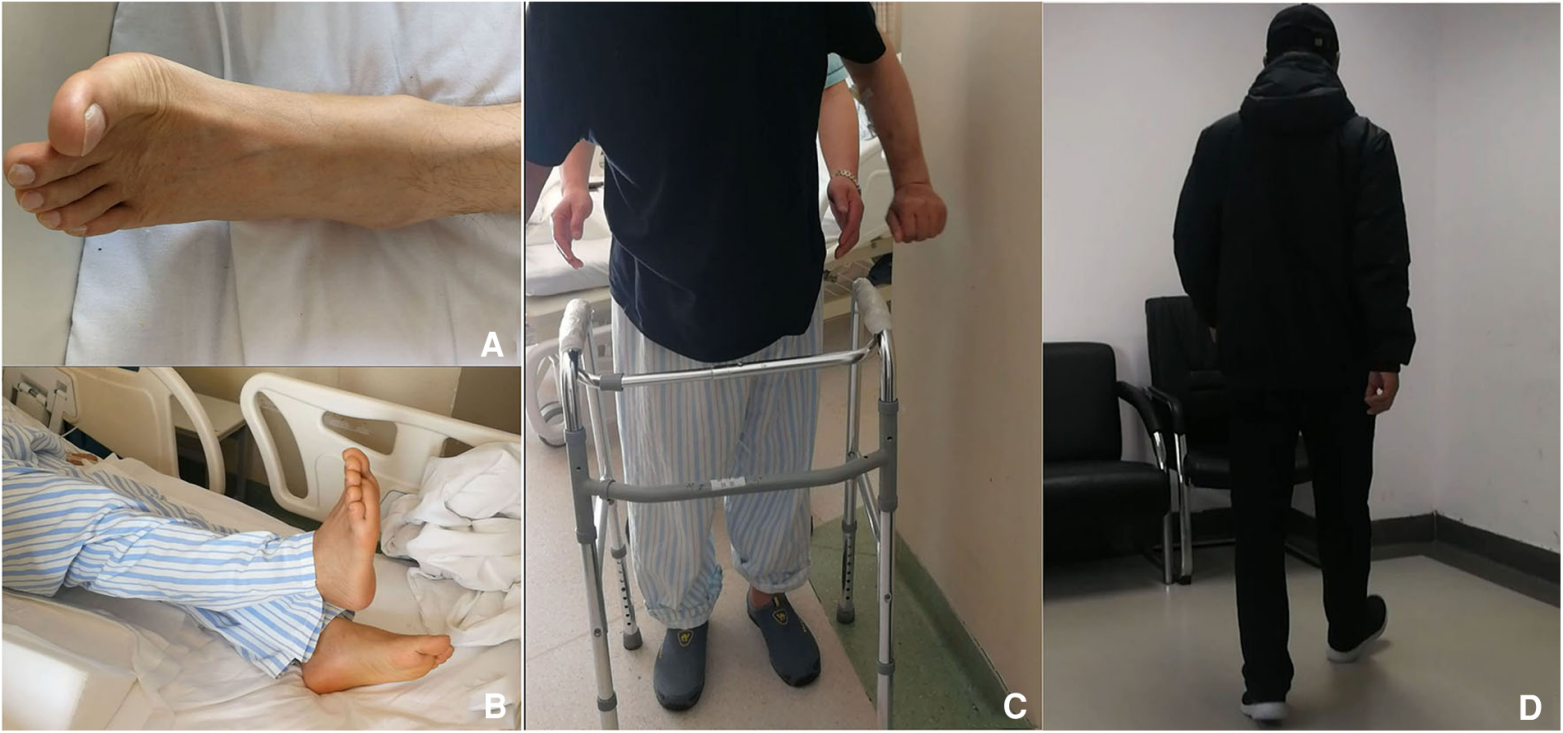
Follow-up postoperatively. (**A**) Dorsal flexion of left big toe could be detected in the surgery morning. (**B**) Right lower limb raising with right ankle at neutral position 1 day postoperatively. (**C**) The patient could walk freely for 5 m 7 days postoperatively. (**D**) Free walking 4 months later.

Neurological function recovered quickly. CT re-examination on 15 May 2019 confirmed fenestration at left T10–11 interlaminar space with complete removal of CLF (Figure 3). After 1 week, the patient could walk a 10-m distance. Low molecular weight heparin doses of 3,000 iu injected subcutaneously per day were continued for another 13 days (about 2 weeks) until the discharge day (8). Aspirin was not restored in fear of SSEH recurrence after consultation of a cardiovascular specialist, left atorvastatin, and isosorbide mononitrate maintained. After 3 months, the patient gained complete recovery (Figure 4). MR re-examination on 26 December 2019 found complete expansion of the dura sac, while intramedullary hyperintensity on sagittal T2-weighted MR imaging sequences could still be detected (Figure 3E). A telephone follow-up on 26 November 2022 revealed normal neurological function without a cardiovascular event since.

## Discussion

Until 2021, only eight thoracic CLF cases were reported in English literature, among them, only one exhibited rapid progression while others exhibited slow progressive process (4) above. All accepted conventional open operations with satisfied results (4) above.

SSEH is commonly characterized by the sudden onset of dorsal local pain, progressive neurological deficits usually within minutes or hours (9). It might result in irreversible neurological sequela if without prompt decompression (5) above. Conservative treatment was limited to those with minimal neurological impairment (10) or significantly rapid improvement (11). Aspirin is a formidable risk factor (12). The preoperative diagnosis of our case was CLF. Emerge of stable blood clots extradural confirmed subacute SSEH intraoperatively (5) above. We preferred the initial symptoms of leg fatigue and paresthesia might be mainly caused by CLF, while the sudden onset of paraplegia and bladder dysfunction came from SSEH.

In 1990, Bracken et al. reported the results of the Second National Acute Spinal Cord Injury Study (NASCIS II protocol) and concluded that patients with acute spinal cord injury (ASCI) treated with MP as a bolus of 30 mg per kilogram of body weight, followed by infusion at 5.4 mg per kilogram per hour for 23 h, improved neurologic recovery when the medication was given in the first 8 h (13). In 1997, Bracken et al. reported the NASCIS III protocol and concluded that patients with ASCI treatment who receive MP within 3 h of injury should be maintained on the treatment regimen for 24 h, and when MP is initiated 3 to 8 h after injury, patients should be maintained on steroid therapy for 48 h (14). Subsequent analysis of the NASCISII and NASCIS III trials has demonstrated potentially serious complications from intravenous high-dose MP, and whether high-dose MP should be accepted as a routine treatment for ASCI patients is still debated (15–17). In 2019, based on a meta-analysis result, Liu et al. concluded that high-dose MP treatment does not make better neurologic recoveries (18). In 2020, Choi et al. reported that ASCI patients who accepted MP administration showed higher complication rates (17) above. In 2022, based on a literature review, Thomas et al. concluded that there were no evidence-based recommendations for pathomechanistically targeted therapies for ASCI patients (16) above. Given the fact that the patient came to our hospital 1 day after the deterioration of his neurological functions, he had missed the 8-h limit to accept high-dose MP. The intravenous drip of 0.5 g of MP was just a clinical trial based on our limited clinical experience without sufficient evidence, which might be regarded as a placebo for the patient but luckily produced neurological improvement. Whether a single 0.5 g dose of MP could be introduced as a routine, however, needs further prospective research.

Aspirin inhibits platelet aggregation (19), it may prevent major vascular complications by inhibiting thrombus formation (20). But there is substantial variability in the perioperative administration of aspirin in patients undergoing noncardiac surgery, both among patients who are already on an aspirin regimen and among those who are not, according to controversial opinions. A study reported by Devereauxj et al. concluded that administration of aspirin preoperatively and throughout the early postoperative period had no significant effect on the rate of a composite of death or nonfatal myocardial infarction but increased the risk of major bleeding (21), while Rychen et al. reported that perioperative continuation of aspirin in elective craniotomies does not seem to be associated with an increased hemorrhagic risk while the rate of thromboembolic events in the continuation group was 3% in comparison to 6% in the discontinuation group (22). Beltrán de Heredia et al. started aspirin-statin therapy postoperatively in patients with silent (asymptomatic) myocardial injury who accepted noncardiac surgery and found rates of mortality and MACCE (major adverse cardiovascular and cerebrovascular events) were still high (23). Our case accepted aspirin-statin therapy 4 years earlier following the diagnoses of coronary atherosclerosis and unstable angina, administration of aspirin was replaced by lower molecular weight heparin 3,000 iu injected subcutaneously per day preoperatively to reduce or avoid the potential risk of major bleeding perioperatively, realizing the function of aspirin in inhibiting thrombus formation could be sustained for 7–10 days (about 1 and a half weeks). No major bleeding occurred intra- and postoperatively, with no occurrence of MACCE at final follow-up. Although only statin therapy was maintained postoperatively.

In the late 1990s, Yeung designed the YESS endoscope to perform endoscopic transforaminal discectomy (24), followed by Hoogland's TESSYS technique (25), and Ruetten's interlaminar technique (26). Endoscopic spine surgery was initially restricted to microdiscectomy for extremely limited contained lumbar disc herniations. With advances in techniques and technology in recent years, the indications for endoscopic spine surgery has expanded to include virtually all types of discs herniations, endoscopic techniques have been utilized for approaching pathology in the cervical, thoracic, and lumbar spine, and endoscopic decompressions have been utilized in the settings of degenerative spinal stenosis, spondylolisthesis, scoliosis, previous fusion, tumor, and infection (27). Recently, PPES was reported to treat thoracic OLF with satisfactory results (28). Theoretically, it was easier to perform decompression using PPES in CLF cases than OLF, as CLF only contains scattered calcified deposits. Not realizing the existence of SSEH preoperatively, we chose PPES for our case with success. Previously, SSEH cases were almost all decompressed by either traditional open surgery (5) above or conservative treatment (11) above. Our procedure implied that PPES might also be suitable for SSEH with normal blood coagulation items. As MP had produced temporary neurological improvement, we might still choose PPES decompression 7 days later in fear of intraoperative bleeding from aspirin administration even if we had detected SSEH preoperatively.

An intramedullary hyperintense signal on T2WI sequences could be detected preoperatively (Figures 1B–D). The abnormalities could still be seen on T2WI MRI sequences 7 months postoperatively, with similar or slightly decreased extent. The signals showed similar MRI exhibitions as those in “White Cord Syndrome (WCS) patients”. Realizing the real WCS entity refers to a rare pathologic condition that affects patients experiencing an unexplained neurologic deficit after spine surgery who exhibited normal intramedullary MRI signals preoperatively (29). Our case gained complete neurologic recovery 3 months postoperatively, meanwhile the pre-existing hyperintense T2WI MRI images did not expand at final MRI re-examination, we do not think our patient belonged to a WCS one. We could not provide exact underlying mechanism because abnormal signals on T2WI sequences still existed 7 months postoperatively, despite the patient having achieved complete neurologic recovery 4 months earlier, and further research is needed.

## Conclusion

SSEH could develop based on CLF and cause serious neurological dysfunctions. PPES technique might be an excellent choice to remove CLF and evacuate SSEH, with minor iatrogenic trauma and good clinical result.

## Data Availability

The raw data supporting the conclusions of this article will be made available by the authors, without undue reservation.
